# Loss of oxidative defense and potential blockade of satellite cell maturation in the skeletal muscle of patients with cancer but not in the healthy elderly

**DOI:** 10.18632/aging.101006

**Published:** 2016-07-23

**Authors:** Joanna Brzeszczyńska, Neil Johns, Alain Schilb, Simone Degen, Martin Degen, Ramon Langen, Annemie Schols, David J Glass, Ronenn Roubenoff, Carolyn A. Greig, Carsten Jacobi, Kenneth CH. Fearon, James A. Ross

**Affiliations:** ^1^ Tissue Injury and Repair Group, Centre for Regenerative Medicine, University of Edinburgh, Edinburgh, Scotland, UK; ^2^ Clinical Sciences (Surgery), University of Edinburgh, Edinburgh, Scotland, UK; ^3^ Novartis Institutes for Biomedical Research Basel, Novartis Pharma AG, CH-4056 Basel, Switzerland; ^4^ Novartis Institutes for Biomedical Research, Cambridge, MA 02139, USA; ^5^ NUTRIM School of Nutrition and Translational Research in Metabolism, Maastricht University, Maastricht, the Netherlands; ^6^ School of Sport, Exercise and Rehabilitation Sciences and MRC-ARUK Centre for Musculoskeletal Ageing Research, University of Birmingham, Birmingham, UK

**Keywords:** cachexia, elderly, satellite cells, oxidative stress

## Abstract

Muscle wasting in old age or cancer may result from failed myofiber regeneration and/or accelerated atrophy. This study aimed to determine from transcriptomic analysis of human muscle the integrity of the cellular stress response system in relation to satellite cell differentiation or apoptosis in patients with cancer (weight-stable (CWS) or weight-losing (CWL)) or healthy elderly (HE) when compared with healthy middle-aged controls (HMA). 28 patients with cancer (CWS: 18 and CWL: 10), HE: 21 and HMA: 20 underwent biopsy of quadriceps muscle. The expression of transcription factors for muscle regeneration (Pax3, Pax7 and MyoD) was increased in CWS and HE compared with HMA (p<0.001). In contrast, the expression of the late myogenic differentiation marker MyoG was reduced in CWS and CWL but increased in HE (p<0.0001). Bax was significantly increased in CWS, CWL and HE (P<0.0001). Expression of the oxidative defense genes SOD2, GCLM, and Nrf2 was decreased in CWS and CWL but increased in HE (p<0.0001). There is evidence for blockade of satellite cell maturation, upregulation of apoptosis and reduced oxidative defense in the muscle of cancer patients. In the healthy elderly the potential for differentiation and oxidative defense is maintained.

## INTRODUCTION

Cancer cachexia is a complex syndrome that affects the majority of cancer patients with advanced disease and impacts adversely on quality of life, treatment tolerance and overall survival [1]. Muscle wasting is a key feature of cancer cachexia and low muscularity has been shown to impact on the strength, mobility, treatment tolerance and independence of the cancer patient [2]. Animal studies have suggested there may be direct or indirect effects of the tumour resulting in muscle atrophy via either decreased myofibrillar protein synthesis, increased protein degradation or a combination of the two [3]. Studies of protein metabolism in cancer in humans are both limited and contradictory [4,5] and have raised the question whether a different mechanism such as failure of myofibre regeneration may be important. In adult humans, muscle mass is lost at ∼1%/year after the age of 30 years [6]. This process is thought to involve a loss of regenerative capacity in the muscle. Nearly 50% of cancer patients are over the age of 70 years and therefore significant pre-morbid age-related muscle loss (sarcopenia) is prevalent in any general cancer population. Equally, many cancer patients have co-morbidities that can result in significant muscle wasting e.g. chronic obstructive pulmonary disease, chronic renal failure or chronic heart failure. Finally, different forms of cancer therapy (chemotherapy, radiotherapy, surgery, targeted therapy) can also induce muscle wasting [1]. Thus, the origin of muscle wasting in any individual cancer patient may be both complex and diverse.

In healthy adult muscle, satellite cells remain in a non-proliferative, quiescent state and are characterized by the co-expression of transcription factors Pax7 and Pax3. Following proliferation, satellite cells maintain the myogenic factors required for myogenic determination including Myf5, MyoD and MyoG, while Pax7 and Pax3 expression is reduced progressively. Activation of muscle specific genes Myf5, MyoD and MyoG is dependent on the Mef2 (myocyte enhancer factor 2) which lacks myogenic activity alone but cooperatively increase the activity of myogenic transcription factors. Subsequently, the cells which begin terminal differentiation start to express factors such as MRF4, myogenin and myosin [7]. Ultimately, these cells fuse to existing damaged muscle fibers or fuse together to form new myofibers during regeneration of damaged skeletal muscle. Loss of skeletal muscle may result from an inability of activated myogenic cells to differentiate and fuse and thereby restore functional fibers due to prematurely impaired myogenesis [8].

Inflammatory cytokines may contribute to the pathology of muscle wasting and defective skeletal muscle regeneration [9]. Epidemiological studies of ageing indicate that IL-6 may also be involved in age-related decline of muscle function [10,11]. In conjunction with TNF- α, IL-6 has also been implicated in the progression of sarcopenia [12]. While increased circulating TNF- α and IL-6 may be markers of systemic inflammation, within muscle it is upregulation of the transcription factor NF-ĸB that may act as a node for inflammatory pathways and promote muscle degeneration [13]. Elevated activity of NF-ĸB has been suggested to suppress MyoD mRNA, leading to dysfunction of skeletal myofibers and muscle loss [14,15] although one study did not indicate a role for NFkB [16]. Non-specific chronic inflammatory processes within the muscle, with excessive production of pro-inflammatory cytokines, may in turn increase the expression of inducible nitric oxide synthase (iNOS) and production of reactive oxygen/nitrogen species (ROS) which can promote muscle weakness and fatigue. ROS can act via redox signaling mechanisms to alter myogenic gene expression, causing protein loss that reduces muscle mass [17]. Secondly, high intracellular ROS also can act via post-translational mechanisms to modify or impair constitutively expressed proteins, causing muscle dysfunction [18]. These two pathways may be regulated independently and can have separate or additive effects on skeletal muscle function and regenerative potential. Here, we focus on the potential role of oxidative stress and apoptosis as a result of inappropriate inflammation in the muscle microenvironment and as a result of mitochondrial damage or inactivation of antioxidant and detoxification systems. The adverse effects of these mechanisms may act through stimulation of nuclear erythroid-2-p45-related factor-2 (Nrf2) and autophagy pathways [19].

There are clear indications that satellite cells play a role in skeletal muscle re-modelling, growth and atrophy [20] and that reduced regrowth of muscle and a reduction in myogenic precursor activity following a period of immobility is associated with aging. It is plausible that inflammation-induced oxidative stress may play a role in the progression of muscle atrophy which subsequently may affect satellite cell activity and muscle fiber remodeling. This study aims to investigate not only the transcriptional differences in satellite cell activity between muscle in healthy aging and patients with cancer but also the mechanisms that may be involved.

## RESULTS

### Patient and volunteer characteristics

The characteristics of the cancer group (C - WS and WL), healthy elderly (HE) and healthy middle aged control (HMA) groups are presented in Table 1. The majority of the cancer patients were male with a mean age of 66 years and an average BMI of 26. Ten patients had lost >5% of their pre-illness weight (cachexia). Most of the cancer patients were Stage II/III disease reflecting their work-up for resectional surgery. Approximately thirty percent had an elevated C-reactive protein. The healthy elderly were more balanced in their sex distribution and had a mean age of 79 yrs. The HE had a mean BMI of 25.4, all were weight-stable and none had an elevated serum C-reactive protein. The healthy middle-aged controls were, on average, nearly two decades younger than the HE and 5 years younger than the cancer patients. There was a predominance of males, average BMI was in the normal range (data not shown), and all were weight-stable.

**Table 1:** Characteristics of all the recruited subjects. The majority of the cancer patients were male (21M:7F)

	Cancer (n=28)		HMA (n=20)	HE (n=21)
	Non cachectic^**^(n=18)	Cachectic^**^(n=10)		
M:F	13:5	8:2	12:8 e	11:10
Age	67 (10.5)	65 (8.1)	61 (7) e	79 (3.6)
Weight	81.8 (14.1)	81.5 (19.3)	73 (12) e	69 (14)
Height	1.69 (0.11)	1.73 (0.1)	1.73 (0.10) e	1.65 (0.09)
BMI	26.6 (4.0)	26 (3.7)		25.4 (3.8)
% WL	6.5 (9.3)	9.3 (7.8)	-	-
LBM (kg)				48.1 (10.5)
FM (kg)				19.1 (6.6)
ASM (kg/m2)	6.4 (0.64)	5.6 (0.4)	7.9 (1.0) e	7.3 (1.3)
% Cachectic^**^	0%	36%		
% Sarcopenic^*^				0%
Cancer type				-
Gastric	3	1		-
Oesophageal	4	4		-
OGJ	5	1		
Pancreas	6	4		
CRP (mg/L)	16.3 (24.8)	26.9 (57.3)		3 (2.5)
CRP >10mg/l	7	2		0

Of the cancer patients, 18 (13M:5F) were non‐cachectic and had a mean age (±SD) of 67 ± 10.5 years and a BMI (±SD) of 26.6 ± 4. Ten (8M:2F) of the cancer patients were cachectic and had lost >5% of their pre‐illness weight, had a mean age (±SD) of 65 ± 8.1 years and an average BMI (±SD) of 26 ± 3.7. Most of the cancer patients were Stage II/III disease reflecting their work‐up for resectional surgery and 30% had an elevated C‐reactive protein. The healthy elderly (HE) (11M:10F) had a mean age (±SD) of 79±3.6 years and a mean BMI (±SD) of 25.4±3.8, all were weight‐stable and none had an elevated serum C‐reactive protein. The healthy middle‐aged controls (HMA) had a mean age (±SD) of 61±7 years. There was a predominance of males, average BMI was in the normal range (data not shown), and all were weight‐stable.

Values are mean and s.d

* Baumgartner's criteria

** Defined by >2% WL and LMM (Low muscle mass)

e = estimated TBC

### Expression of satellite cell activation and myogenic differentiation

**Figure 1:**
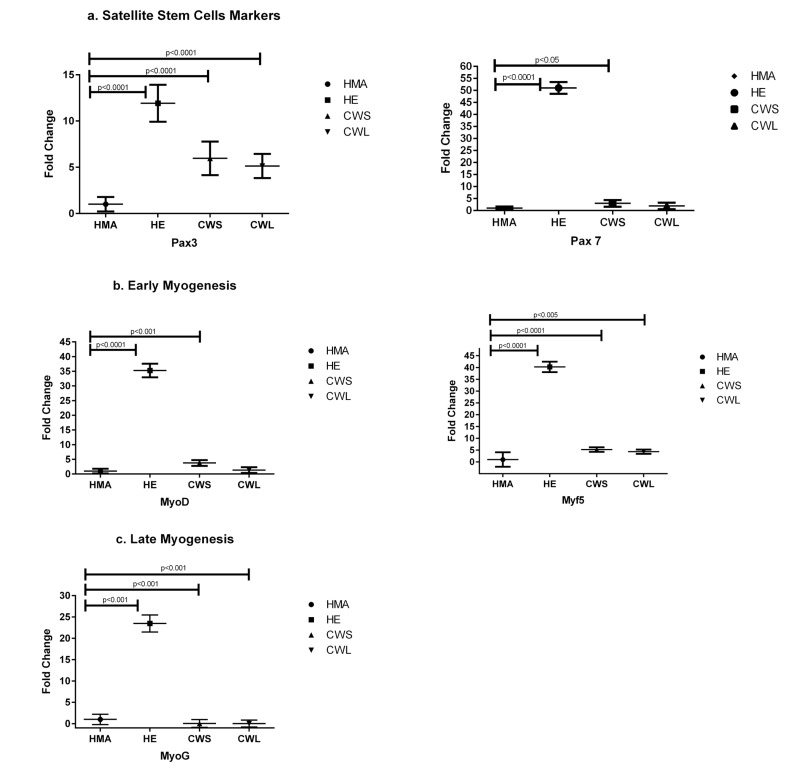
Fold change (±SD) in mRNA expression of genes related to (**a**) satellite cell markers (Pax3 and Pax7), (**b**) myoblast proliferation/early differentiation (MyoD and Myf5) and (**c**) late myocyte/myotube differentiation (MyoG) in the quadriceps muscle of C, HE and HMA groups: HMA (n=22), HE (n=17), CWS (n=18), CWL (n=10). Fold-change is relative to HMA participants. Statistical analysis was carried out on deltaCT values. Q-PCR results show upregulation of mRNA levels of Pax3 (p<0.0001 in HE, CWS and CWL) compared with HMA and Pax7 (p<0.0001 in HE; p<0.05 in CWS) compared with HMA. Expression of early myogenesis markers was increased: MyoD (p<0.0001 in HE and p<0.001 in CWS) and Myf5 (p<0.0001 in HE and CWS, P<0.005 in CWL) compared with HMA. The expression of MyoG is upregulated in HE (p<0.0001) compared with HMA and reduced in CWS and CWL (p<0.0001) compared with HMA.

The expression of the muscle transcription factor Pax3 was significantly higher in both cancer groups and HE compared with HMA (Fig. 1a). A similar pattern was observed for Pax7 but this was only elevated in CWS and not CWL compared with HMA. There was increased expression of markers of early myogenesis (MyoD and Myf5) in both HE and CWS when compared with HMA (Fig. 1b). Myf5 was also increased in CWL compared with HMA (Fig. 1b). When compared with HMA there was elevated expression of myogenin (MyoG) in the HE possibly indicating the ongoing potential for myogenic fusion (Fig.1c). In contrast, myogenin was down- regulated in muscles of both CWS and CWL.

### Expression of pro-inflammatory cytokines and NFĸB

**Figure 2:**
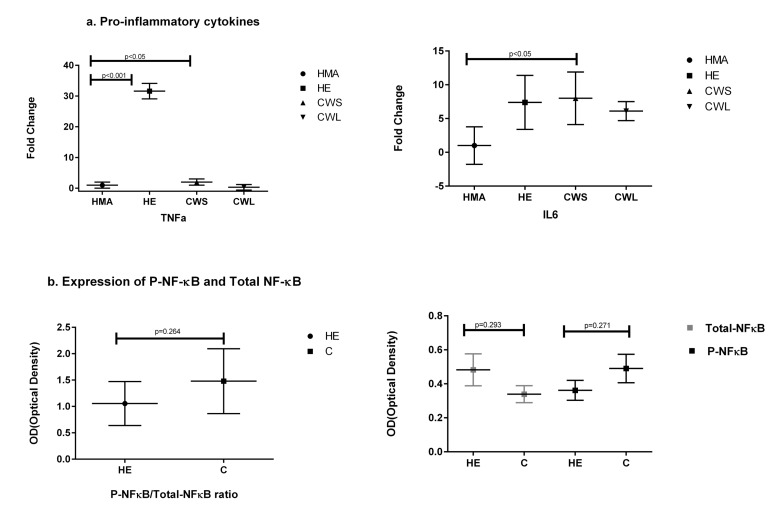
Fold change (±SD) in mRNA expression of (**a**) genes of the pro-inflammatory cytokines TNF and IL-6 in the Quadriceps muscle of C (all cancer patients), HE and HMA groups: HMA (n=22), HE (n=15), CWS (n=16), CWL (n=8). Fold-change of mRNA expression is relative to HMA participants. Statistical analysis was carried out on deltaCT values. Q-PCR shows upregulation of mRNA expression of TNF-α in HE (p<0.001) and in CWS (p<0.05) and IL-6 in CWS (p<0.05) compared with HMA and (**b**) illustrates protein analysis showing total NF-ĸB and pNF-ĸB in the Quadriceps muscle. Data are presented as P-NF-ĸB/total NFĸB ratio which was not significantly different for HE compared with C. The mean protein expression ± SEM were analysed for HE (n=8) and C (n=9) groups for P-NF-ĸB and C (n=9) and HE (n=9) groups for NF-ĸB. Neither total NF-ĸB nor pNF-kB were significantly different between the HE and C groups (p=0.291 and p=0.271 respectively).

When compared with HMA, message for TNF-α was highly expressed in the muscle of HE (30 fold) and to a lesser extent in the muscle of CWS (3 fold) (Fig. 2a). IL-6 message was significantly increased only in CWS compared with HMA (Fig. 2a). Samples for Western blot analysis were not available for the HMA group and for only a subgroup of the cancer patients (n=9). In view of the small sample size, CWS and CWL were amalgamated into one cancer (C) group. Using Western blot analysis, the p-NFĸB/total NFĸB ratio was not significantly different between the HE and C groups. Similarly, levels of pNF-ĸB and total NF-ĸB protein levels were not significantly elevated in the muscle of HE compared with the C group (Fig. 2b).

### Expression of apoptotic factors, autophagy and oxidative stress defense genes

**Figure 3:**
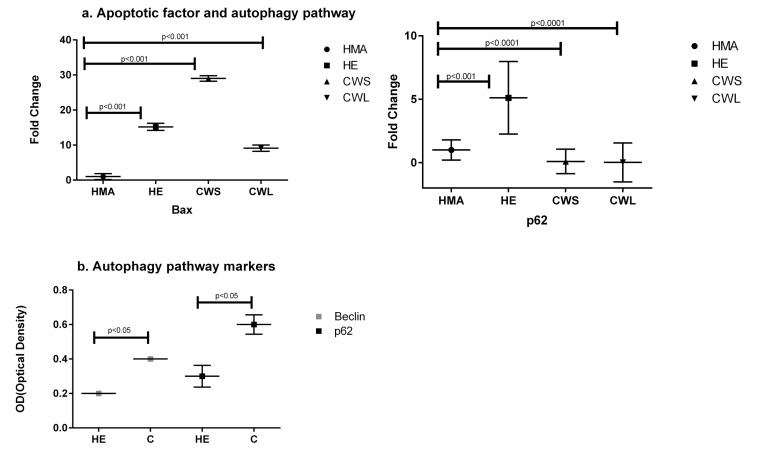
Fold change (±SD) in expression of mRNA for (**a**) the apoptotic factor Bax and the autophagy-related Sequestosome 1 (p62/SQSTM1) in the Quadriceps muscle of C, HE and HMA groups: HMA (n=22), HE (n=20), CWS (n=18), CWL (n=10). Fold-change of mRNA expression is relative to HMA. Statistical analysis was carried out on deltaCT values. Q-PCR results show upregulation of pro-apoptotic Bax (p<0.001) in HE and C (CWS and CWL) muscle compared with HMA. Expression of mRNA for p62/SQSTM1 is upregulated in HE (p<0.001) and downregulated in CWS and CWL (p<0.0001) and (**b**) Western blot analysis indicates activation of the degradation pathway (Beclin and p62/SQSTM1) in the Quadriceps muscle of C compared with the HE group. Data are means ± SEM from C (n=9) and HE (n=8) groups for p62 and C (n=9) and HE (n=9) for Beclin. The analysis demonstrates significant upregulation of these proteins in muscle from C compared with HE (p<0.05).

**Figure 4:**
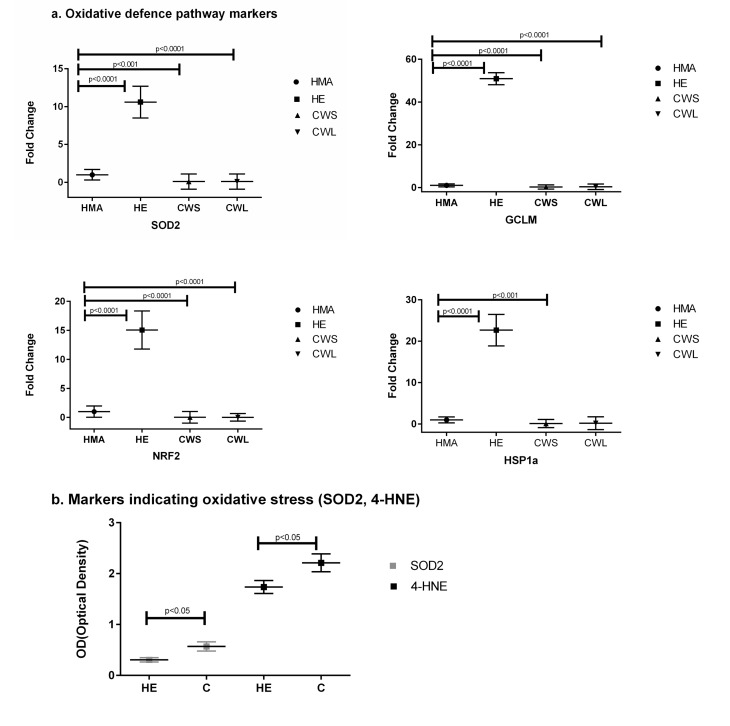
(**a**) Fold change (±SD) in mRNA expression of the oxidative defense genes SOD2, GCLM, and Nrf2 and the heat shock protein HSP1a in the Quadriceps muscle of C, HE and HMA groups: HMA (n=22), HE (n=17), CWS (n=18), CWL (n=9); Fold-change of mRNA expression is relative to HMA participants. Statistical analysis was carried out on deltaCT values. The mRNA expression of the oxidative defense gene SOD2 is increased in HE (p<0.0001) but decreased in CWS (p<0.001) and CWL (p<0.0001) compared with HMA. Expression of GCLM is also increased in HE (p<0.0001) but decreased in CWS (p<0.0001) and CWL (p<0.0001) compared with HMA. Nrf2 is decreased in CWS and CWL but increased in HE when compared with HMA (p<0.0001) and HSP1a is decreased in CWS (p<0.001) but increased in HE (p<0.0001) when compared with HMA. (**b**) Western Blot analysis of the cellular defensive pathway (SOD2, 4-HNE) in the Quadriceps muscle of C and HE groups. Data are means ± SEM from C (n=10) and HE (n=8) groups for SOD2 and C (n=9) and HE (n=8) for the hydroxyalkenal 4-HNE. The analysis demonstrates upregulation of both SOD2 and 4-HNE in C compared with HE (p<0.05).

The expression of the pro-apoptotic gene Bax (Fig. 3a) was significantly increased in CWS, CWL and HE compared with HMA (P<0.001). In HE, CWS and CWL, expression of message for Sequestosome 1 (p62/SQSTM1), p62, which is an indicator of oxidative stress, was significantly different from HMA (Fig. 3a). Both Beclin and p62 protein expression were significantly increased in cancer compared with HE. Expression of message for the oxidative defense genes SOD2, GCLM, Nrf2 and HSP1a (Fig. 4a) was increased in HE when compared with HMA (P<0.0001). Increased nitrosylation (4-HNE) and high SOD2 levels (indicating elevated oxidative stress) were observed in the muscle of cancer patients compared with those from HE (Fig. 4b). There is a well recognised divergence between the transcriptome and the proteome, and while message for SOD2 was significantly higher in healthy elderly (HE) compared with the HMA group (Fig. 4a), the level of the translated protein SOD2 was significantly higher in cancer patients compared with healthy elderly (HE) (Fig. 4b). In addition, the alpha,beta-unsaturated hydroxyalkenal, 4-HNE, produced by cellular lipid peroxidation and indicating high oxidative stress, was also significantly higher in cancer patients compared with healthy elderly (HE) (Fig. 4b).

## DISCUSSION

In contrast to some studies [20], the present study has shown that the expression of transcriptional factors responsible for early aspects of muscle regeneration is higher in the skeletal muscle of the elderly and both cancer patient groups than in muscle from middle-aged controls. In particular, the markedly increased levels of Pax3 and particularly Pax7 in the elderly suggested a significant stimulation of regenerative activity. The very low expression of MyoG (late marker of differentiation) in the cancer cohort (both CWS and CWL) supports the concept that myogenic differentiation is impaired prematurely in cancer patients and that activated myoblasts are unable to proceed towards terminal differentiation and fibre remodelling [8]. Conversely, in the elderly muscle, myogenic capability appeared to be preserved. In contrast, middle-aged healthy controls have muscle which has not been subjected to stress or injury and appears to remain in a quiescent state without ongoing regeneration.

The need for regeneration will be increased in the presence of cell injury or active apoptosis. A previous study suggested the possibility of apoptosis in the muscles of humans with cancer cachexia [26]. In the present study, Bax expression was increased in the HE and cancer groups suggesting the induction of apoptosis. The Nrf2-Keap1 pathway is thought to be central to the induction of potent cellular antioxidant and detoxification systems that protect against the induction of apoptosis [27]. In this context, down-regulation of Nrf2, specifically in the cancer patients (Fig. 4a), may be important as down-regulation of Nrf2 has been associated with increased apoptosis [19]. In addition, an increase in nitrosylation and oxidative defense proteins was detected in the muscle from cancer patients (Fig. 4b). These findings support the concept that muscle wasting in cancer and the elderly may, in part, be due to continuous oxidative stress and apoptosis [17].

Pro-inflammatory cytokines have been strongly implicated in the muscle wasting associated with both cancer and old age. In the present study, there was evidence of mild systemic inflammation in the cancer group, with approximately one third having an elevated circulating C-reactive protein, but not in the healthy elderly. Within the muscle compartment, there was evidence of increased expression of pro-inflammatory cytokines (TNF-alpha) in the healthy elderly and to a lesser extent in the CWS patients when compared with the middle-aged controls (Fig. 2a). The expression of IL-6 was also significantly elevated in the CWS group compared with the HMA group (Fig. 2a) suggesting an ongoing inflammatory process. Although there was no evidence of significant activation of NF-ĸB (pNF-ĸB) or an increase in the expression of NFkB protein in the muscle of cancer patients (Fig. 2b), this may be be due to the smaller sample sizes available for protein analyses masking a very low level of activation. Cytokines such as TNF-α are known to be direct inducers of muscle cell death, while NF-kB is known to block MyoD and Mef2 and inhibit muscle remodeling [14–16].

Cellular stress can influence the regulation of autophagy and apoptosis pathways [28]. Elimination of unwanted ROS is therefore of great importance for cell survival. In the present study, no evidence of significant transcriptional activation of cellular defenses (SOD2) was found in the muscle of the cancer patients. However, the increased levels of SOD2 protein and nitrosylation (indicated by expression of the unsaturated hydroxyalkenal 4-HNE) suggesting oxidative stress, were significantly higher in cancer patients (C) compared with healthy elderly (HE) (Fig. 4b). This finding directly reflects oxidative stress in the tissues. The lack of a transcriptomic response may indicate impaired defenses against oxidative stress which may contribute to a reduction in muscle regeneration.

It has been suggested that autophagy is increased in muscle of cancer patients and contributes to increased protein degradation and muscle wasting [29]. In stressful conditions or in tissue with increased protein misfolding and aggregation, autophagy has an important role in eliminating protein aggregates. Under normal conditions p62 is degraded through autophagy. In the present study, the expression of p62 and Beclin1, which participates in autophagy [30], was increased in cancer patients compared with the healthy elderly (Fig. 3b). It is possible that in cancer patients mitochondrial-derived ROS promote skeletal muscle autophagy [19] and, in a murine system, a potential role for mitochondrial dys-function and oxidative stress in autophagy-related pathology has been suggested [31]. Oxidative stress has also been reported to activate nuclear NF-κB as a result of p62 upregulation and TNF receptor-associated factor 6 (TRAF6) complex formation leading to antioxidant-defense system activation [32] and, as discussed above, inhibition of muscle remodelling [14–16].

### Strengths and weaknesses of study

One limitation of the present study was the relatively small sample size. An additional limitation was that the healthy middle-aged participants were recruited to provide reference data for mRNA analysis and we did not obtain muscle biopsies from HMA for protein analysis. We were unable therefore to confirm that the protein signatures obtained in the elderly and cancer groups were different from the healthy middle-aged. One weakness of working with human samples that are not longitudinal is that the samples represent a snapshot of the current status of the patient at a certain time point of the disease, which makes the correlation of the transcriptome with the proteome challenging, as there is a time delay between the change already observed in gene-expression and any subsequent influence on the proteome. Overall, our analysis can be treated as a pilot study for more definitive studies with larger numbers of participants and better defined cohorts, which will allow more robust comparisons at both the transcriptome and proteome levels. In addition, the sampling and analysis of different tissues (e.g. serum, urine, target tissue) might aid further interpretation of the data from such a complex human paradigm.

## MATERIALS AND METHODS

### Patients/volunteers

All subjects gave written informed consent prior to entry into the study. All procedures were approved by the NHS Lothian local research Ethics Committee. The study conformed to the standards set by the Declaration of Helsinki.

Cancer (C): 28 cancer patients were identified via the upper gastrointestinal cancer multi-disciplinary team (MDT) at the Royal Infirmary, Edinburgh, UK. Patients had newly diagnosed potentially resectable cancer.

Healthy Elderly (HE): 29 volunteers aged >75 years were recruited using advertisements in local newspapers and were defined as healthy on the basis of their responses to previously published health selection criteria [21]. None were engaged in any form of physical training. To exclude patients with age-related sarcopenia, volunteers were screened using body composition analysis by DEXA scanning and only those patients who did not fulfil the criteria for sarcopenia (n=21) were included.

Healthy Middle Age Controls (HMA): 20 volunteers were recruited by using advertisements in local newspapers. It was confirmed that volunteers had no recent weight loss or any common disease associated with cachexia (cancer, severe COPD, congestive heart failure, active infectious disease). Individuals taking hormones or continual oral steroids were excluded.

### Calculation of weight loss

Pre-morbid weight was recalled by the cancer patients and verified where possible from the medical notes. Individual weight-loss was calculated and expressed as a percentage of pre-morbid body weight loss. Patients were considered to be weight-losing (WL) if they had lost more than 5% of their pre-morbid weight [23]. The second group of cancer patients were considered as the weight-stable (WS) group.

### Measurement of C-reactive protein

C-reactive protein was measured in serum using an automated hospital-based technique (Abbott TDX).

### Muscle biopsy

A Bergstrom needle muscle biopsy was obtained from the lateral mass of the quadriceps either under local anaesthetic (HE and HMA) or immediately at the start of general anaesthesia and prior to the start of open abdominal surgery (C). The biopsy was cleaned of gross blood contamination and snap frozen in liquid nitrogen and stored at −80°C.

### DEXA body composition analysis (HE and HMA)

Dual-energy X-ray absorptiometry (DPX-L; Lunar Radiation Corp) was used to measure different body compartments (i.e. fat mass, lean mass, and bone mineral content).

### Total RNA isolation

Tissue was homogenised in Qiazol (Qiagen, UK) reagent using a Polytron PT1200E (Kinematica AG, Switzerland). Total RNA was extracted using miRNEasy columns (Qiagen, UK) as directed by the manufacturer with an on-column DNAse digestion step. RNA was quantified using the Nanodrop instrument (Labtech Intl, UK). Quality and purity of RNA was examined using 260/280 and 260/230 ratios. The Agilent bioanalyzer (Agilent, UK) was used to assess RNA integrity using previously published protocols [24]. All samples had 260/280 ratios above 1.8, and RIN scores above 7.5.

### Quantitative Real-Time Polymerase Chain Reaction (QRT-PCR)

The High Capacity RNA-to-cDNA kit (Applied Biosystems, UK) was used to convert 1μg of RNA to cDNA following the manufacturer's directions. The quantitative validation of the expression of selected genes was performed by QRT-PCR (Applied Biosystems StepOne Real-Time PCR Systems) using custom PrimerDesign primers and applying the Sybre Green PCR master mix (Applied Biosystems, Foster City, CA, USA), following the manufacturer's protocol. Reactions were run in triplicate on a StepOne Plus instrument (Applied Biosystems, UK*).* Running conditions were 95°C 10 minutes followed by 40 cycles of 95°C 15 seconds and 60°C 60 seconds. Amplification was performed for each cDNA (20ng) sample in triplicate. The fold change in expression of the target gene relative to the internal control gene (SDHA and CYC1) was assessed. QRT-PCR data were presented as the fold-change in gene expression normalized to average value of two common endogenous reference genes and relative to the control (HMA muscles). See Table 2 for primers for QRT-PCR.

**Table 2:** Sequences for the quantitative real-time PCR primers (PrimerDesign, UK) designed to support the MIQE guidelines: minimum information for publication of quantitative real-time PCR

Gene name	Forward Primer	Reverse Primer	Amplification size (bp)
Pax 3	TCTTACCAGCCCACATCTATTC	TGGAAGGAATCGTGCTTTGG	109
Pax 7	TGTGCCCTCAGGTTTAGTGA	CCGTCGTCCTCCTTCTTGT	99
MyoD	CGCCTGAGCAAAGTAAATGAG	GCCCTCGATATAGCGGATG	117
Myf5	CACCTCCAACTGCTCTGATG	TAAGGAGTTTTTATCTGTGGCATATAC	127
MyoG	GCCCTGATGCTAGGAAGCC	CTGAATGAGGGCGTCCAGTC	110
TNF	AGGTTCTCTTCCTCTCACATAC	ATCATGCTTTCAGTGCTCATG	82
IL6	GCAGAAAACAACCTGAACCTT	ACCTCAAACTCCAAAAGACCA	116
Bax	ATGGAGCTGCAGAGGATGAT	CAGTTGAAGTTGCCGTCAGA	101
P62	ACCATCCAGTATTCAAAGCATCC	AAGAGGGGCACGCAGAAG	70
SOD2	CGACCTGCCCTACGACTAC	AACGCCTCCTGGTACTTCTC	132
GCLM	GGAATTATCAAATCAAAAGGCTACATT	TTTTTACACATCTCAATTTTCTCTCAT	120
NRF2	CCCAGCACATCCAGTCAGA	CAGTCATCAAAGTACAAAGCATCT	91
HSP1	GCGTGATGACTGCCCTGAT	GTTGTCGGAGTAGGTGGTGAA	80
Mef2	GCAGGAATTTGGGAACTGAGC	GGAACAGCTTGTTGGTGCTG	297

### Identification of suitable reference genes

Given the progressive nature of the transcriptional dysregulation phenotype in the C, HMA and HE groups it was critical to determine which genes were most suitable for normalisation in different muscle groups. We therefore utilised the geNorm Housekeeping Gene Selection Kit (PrimerDesign) to evaluate 12 commonly used housekeeping genes in 3 different muscle groups. Reference genes tested were 18S (18S ribosomal RNA subunit), β-Actin (beta-actin), ATP5b (ATP synthase subunit 5b), B2M (beta-2 microglobulin), Top1 (topoisomerase 1), CYC1 (cyclin D1), EiIF4a2 (eukaryotic initiation factor 4a2), GAPDH (glyceraldehyde-3-phosphate dehydrogenase), RPL13a (ribosomal protein L13a), SDHA (succinate dehydrogenase complex, subunit A), UBC (ubiquitin C) and YHWAZ (phospholipase A2). The geNorm output ranked the candidate reference gene according to their expression stability (M). Using this approach we identified 2 housekeeping genes for disease and non-disease conditions and we determined whether the optimal reference genes identified for use in degenerating muscles were also suitable for use as a reference in HMA muscles. Therefore, we identified SDHA and CYC1 as being the most stably expressed genes in healthy elderly and cancer muscles as well as in healthy middle age control (data not shown). These two reference genes were taken for the following analysis.

### Tissue preparation for protein extraction

Skeletal muscle tissue was minced and ground on dry ice. Aliquots were weighed using an analytical balance (Mettler Toledo) and stored at −80°C until use.

### Protein extraction

Proteins were extracted from pulverized human skeletal muscle tissue by homo-genizing the samples in the Precellys 24 system. Briefly, 300 μl of PhosphoSafe Extraction Reagent (Millipore) was added to minced and ground human skeletal muscle tissue (8mg) in Precellys 24 lysing kit tubes. Tissue was further homogenized using the high-throughput homogenizer Precellys 24, for 10s.). After incubation on ice for 5 minutes, the lysates were spun at 800xg for 5 min at 4°C. Supernatants were transferred into new tubes and spun for another 12 min at 1600xg at 4°C. Pellets (insoluble fraction) were stored at −80°C until further us. Supernatants were collected and protein concentrations measured using the BCA Protein Assay Kit (Pierce) with BSA as a standard. Afterwards, phosphatase inhibitor cocktail (Roche) was added and the samples were stored at −80°C until further use.

### Western blots

10 μg of human skeletal muscle protein extracts (see above) in reducing Laemmli SDS sample buffer were boiled for 5 min at 95°C and then separated by SDS-PAGE on 4-20% gradient gels (Bio-Rad, Cressier, Switzerland), blotted to Nitrocellulose membranes (Bio-Rad) using the Trans-Blot Turbo Transfer System (Bio-Rad), blocked for 1h in blocking buffer (5% non-fat milk in Tris-buffered saline 0.05% Tween-20), incubated overnight with primary antibody, rinsed, and incubated for 1h with peroxidase-conjugated goat anti-rabbit IgG at room temperature. Blots were developed using ECL (Roche, Rotkreuz, Switzerland) or SuperSignal West Femto substrate (Thermo Scientific, Wohlen, Switzerland) and exposed to Kodak film (Kodak, Rochester, NY, USA). Antibodies: p62/SQSTM1 (SIGMA, Saint Louis, MO, USA), Beclin-1 (clone D40C5), NF-κB p65 (clone D14E12), phospho-NFκB p65 (Ser536) (clone 93H1) (Cell Signaling Technologies, Danvers, MA, USA), Superoxide Dismutase-2 (MnSOD) (clone DD-17) (SIGMA, Saint Louis, MO, USA), goat polyclonal 4-Hydroxynonenal (4-HNE) (Millipore, Billerica, MA, USA). Western blots were analyzed densitometrically using ImageJ software version 1.45 (NIH, Bethesda, MD, USA; http://rsbweb.nih.gov/ij). Band intensity of each sample was normalised to coomassie blue stained gels.

### Statistical analysis

For statistical analysis the SPSS package (IBM, UK) was utilized. As there were four participant groups and the bio-markers are all continuous we have used analysis of variance (ANOVA) to determine if there is evidence of differences across the groups. Statistical significance of the Q-PCR results was assessed by the ANOVA with Tukey HSD correction to evaluate the differences between means. This analysis was carried out on the deltaCT values before calculation of the transcript copy number (fold change). The graphical representation of the QPCR results represents the calculated transcript copy number and the statistics shown in the graphs represents analysis based on the deltaCT values. Results were considered significant at P<0.05. Statistical analysis of the Western blot data was carried out using Graphpad Prism6 software package (GraphPad Software, Inc., La Jolla, CA, USA). Statistical significance of the results was assessed by the Mann Whitney Test as described by Eaton et al [25].
